# The effect of surgical and non-surgical weight loss on N-terminal pro-B-type natriuretic peptide and its relation to obstructive sleep apnea and pulmonary function

**DOI:** 10.1186/s13104-016-2241-x

**Published:** 2016-09-13

**Authors:** Anne-Marie Gabrielsen, Torbjørn Omland, Mette Brokner, Jan Magnus Fredheim, Jens Jordan, Sverre Lehmann, May Brit Lund, Jøran Hjelmesæth, Dag Hofsø

**Affiliations:** 1 Morbid Obesity Center, Vestfold Hospital Trust, PO Box 2168, 3103 Tønsberg, Norway; 2 Department of Internal Medicine, Vestfold Hospital Trust, Tønsberg, Norway; 3 Division of Medicine, Akershus University Hospital, Lørenskog, Norway; 4 Institute of Clinical Medicine, Center for Heart Failure Research, University of Oslo, Oslo, Norway; 5 Department of Clinical Chemistry, Vestfold Hospital Trust, Tønsberg, Norway; 6 Institute for Clinical Pharmacology, University of Hannover, Hanover, Germany; 7 Department of Thoracic Medicine, Haukeland University Hospital, Bergen, Norway; 8 Department of Respiratory Medicine, Oslo University Hospital, Oslo, Norway; 9 Department of Endocrinology, Morbid Obesity and Preventive Medicine, Institute of Clinical Medicine, University of Oslo, Oslo, Norway; 10 Department of Clinical Science, University of Bergen, Bergen, Norway

**Keywords:** Obesity, Natriuretic peptides, Sleep apnea, Respiration

## Abstract

**Background:**

Obesity is a major risk factor for obstructive sleep apnea, impaired pulmonary function and heart failure, but obesity is also associated with paradoxically low levels of serum N-terminal pro-B-type natriuretic peptide (NT-proBNP). In subjects with severe obesity undergoing weight loss treatment, we assessed the associations between changes in severity of obstructive sleep apnea, pulmonary function and serum NT-proBNP levels.

**Methods:**

One-year non-randomized controlled clinical trial. Participants, 69.6 % women, mean (SD) age 44.6 (10.8) years and body mass index (BMI) 45.1 (5.6) kg/m^2^, underwent gastric bypass surgery (n = 76) or intensive lifestyle intervention (n = 63), resulting in 30 (8) % and 8 (9) % weight loss, respectively. The reference group included 30 normal weight, healthy, gender and age matched controls. Sleep recordings, arterial blood gases, pulmonary function and blood tests were assessed before and 1 year after the interventions.

**Results:**

NT-proBNP concentrations increased significantly more after surgery than after lifestyle intervention. The post intervention values in both groups were significantly higher than in a normal weight healthy reference group. In the whole study population changes (∆) in NT-proBNP correlated significantly with changes in both BMI (r = −0.213) and apnea hypopnea index (AHI, r = −0.354). ∆NT-proBNP was, independent of age, gender and ∆BMI, associated with ∆AHI (beta −0.216, p = 0.021). ∆AHI was, independent of ∆BMI, significantly associated with changes in pO_2_ (beta −0.204), pCO_2_ (beta 0.199), forced vital capacity (beta −0.168) and forced expiratory volume first second (beta −0.160).

**Conclusions:**

Gastric bypass surgery was associated with a greater increase in NT-proBNP concentrations than non-surgical weight loss treatment. Reduced AHI was, independent of weight loss, associated with increased NT-proBNP levels and improved dynamic lung volumes and daytime blood gases.

*Clinical Trial Registration* ClinicalTrials.gov NCT00273104, retrospectively registered Jan 5, 2006 (study start Dec 2005)

**Electronic supplementary material:**

The online version of this article (doi:10.1186/s13104-016-2241-x) contains supplementary material, which is available to authorized users.

## Background

Obesity is a major risk factor for obstructive sleep apnea (OSA), impaired pulmonary function and heart failure [1–4]. Fat deposits narrow upper airways while abdominal fat masses decrease tracheal tension, thus, increasing upper airway collapsibility [5]. Obesity also restricts diaphragm movement and causes alveolar and airway closure at the lung base, thus perturbing respiratory physiology and blood gases [6–8]. Increased heart failure risk may be mediated through obesity-associated arterial hypertension, volume expansion, dyslipidemia, and insulin resistance [9, 10]. The presence of OSA and impaired pulmonary function may further exacerbate cardiovascular risk and symptom burden.

ProBNP produced in ventricular cardiomyocytes is processed to the N-terminal peptide (NT-proBNP) and biologically active BNP. Circulating BNP and NT-proBNP levels are established heart failure biomarkers [11–13]. In spite of the cardiac overload, circulating NT-proBNP/BNP concentrations are paradoxically reduced in obesity [14, 15], and increase following either weight loss induced by bariatric surgery [16, 17] or intensive lifestyle intervention [18]. Impaired natriuretic peptide release and increased degradation of BNP through adipose natriuretic peptide clearance receptors [19] and neprilysin [20] have been reported. Moreover, adipose natriuretic peptide clearance receptor expression decreases with weight loss [21]. Although NT-proBNP is not cleared by NPR-C receptors or neprilysin, there is still an inverse association between body mass index (BMI) and NT-proBNP [15]. Natriuretic peptides counteract cardiac overload through natriuresis and vasodilatation. They also augment lipid mobilization and muscular oxidative capacity, thereby ameliorating obesity-associated metabolic disease [22–28]. Finally, natriuretic peptide receptors are expressed in the lung, and natriuretic peptide infusion elicits bronchial dilatation [29]. While relative natriuretic peptide deficiency could link cardiometabolic and pulmonary disease in obesity, data concerning the associations between OSA severity and pulmonary function, blood gases and NT-proBNP is scarce [30].

We have previously shown that improvements in obstructive sleep apnea [5], pulmonary function and blood gases [31], and obesity related cardiovascular risk factors [32, 33] in subjects with morbid obesity following surgical and non-surgical weight loss are mediated through weight loss and not by the surgical procedure per se. This ancillary study assesses the impact of surgical and non-surgical weight-loss, obstructive sleep apnea, and pulmonary function on circulating NT-proBNP. Given the hemodynamic stress imposed by OSA and pulmonary disease, we hypothesized that improvement in OSA and pulmonary function would counterbalance the expected increase in NT-proBNP during weight loss. Furthermore, we assessed the associations between apnea hypopnea index (AHI) and pulmonary function during weight loss.

## Methods

### Subjects and intervention

The MOBIL study (ClinicalTrials.gov identifier NCT00273104) was conducted at Vestfold Hospital Trust, Norway, between December 2005 and June 2009. Patients in the surgery group underwent laparoscopic Roux-en-Y gastric bypass surgery and were examined by a surgeon after 6 weeks and assessed by a dietician quarterly. Moreover, the subjects in both treatment groups were seen by an internist every half a year, in addition to a dietician when required. The subjects in the lifestyle group were referred to a rehabilitation center (Evjeklinikken) specializing in the care of morbid obesity. The 1-year lifestyle program aimed to induce a weight loss of at least 10 %, and composed of four stays at the center, with a total of a 7 weeks stay. The daily program was divided between physical activity (3–4 h) and different psychosocial interventions involving a medical doctor, a nutritionist, a physiotherapist and a trained nurse. Details concerning the interventions have been published previously [32]. For analyses of NT-proBNP the subjects with morbid obesity were compared with a reference group of 30 normal weight controls (67 % female), mean BMI (SD) 22.7 (1.5) kg/m^2^, mean age (SD) 42.6 (8.5) years, recruited among employees at Vestfold Hospital Trust.

### Examinations and definitions

Daytime arterial blood gases, pulmonary function tests, sleep registrations and blood samples were carried out at baseline and 1 year after intervention. All subjects were examined by a physician. Weight, height, medical history, including smoking (pack/years), medications and electrocardiograms were recorded [31]. Asthma was defined as physician-diagnosed asthma, and chronic obstructive pulmonary disease was defined as forced expiratory volume first second (FEV1)/forced vital capacity (FVC) <0.7 [34].

### Sleep recordings

Sleep recordings were performed using Embletta™; a nine channel portable somnograph including pulse oximetry. An apnea was defined as a 90 % or more reduction in baseline nasal airflow lasting at least 10 s. A hypopnea was defined as a 50–90 % reduction in pre-event nasal airflow lasting ≥10 s accompanied by at least a 3 % drop in oxygen saturation. AHI was defined as the total number of apneas and hypopneas (events) per hour. Oxygen desaturation index was defined as the number oxygen desaturations (≥3 % drop) per hour. Obstructive sleep apnea (OSA) was defined as being ≥5 events per hour during sleep. OSA was categorized into mild (AHI 5 to <15 events/hour), moderate (AHI 15 to <30 events/hour) and severe (AHI ≥ 30 events/hour) (5). Scoring rules were in accordance with the 2007 American Academy of Sleep Medicine manual for scoring sleep [35]. To assess the effect of moderate/severe OSA in this study, we dichotomized the subjects into high AHI (≥15 events/hour) and low AHI (<15 events/hour).

Patients with AHI ≥ 15 events/hour, or AHI 5 to <15 events/hour and symptoms of sleep apnoea, were offered a CPAP (Continuous positive airway pressure—Autoset spirit, ResMed), while patients with additional daytime hypercapnia (PCO_2_ > 6.0 kPa) were offered a bilevel PAP (VPAP 4, ResMed). These subjects were followed at their outpatient clinics.

### Pulmonary function measurements

Pulmonary function measurements included dynamic spirometry, static lung volumes and gas diffusion capacity. These tests were carried out by two experienced nurses according to the guidelines recommended by the ATS-ERS task force [36–38]. Recorded variables were: FVC (forced vital capacity), FEV1 (forced vital capacity the first second first second), FEV1/FVC, TLC (total lung capacity), VC (vital capacity), IC (inspiratory capacity), FRC (functional residual capacity), ERV (expiratory reserve volume), RV (residual volume), DLCO (diffusion capacity of carbon monoxide) and DLCO/VA (DLCO/alveolar volume). The Jaeger Master Lab (Eric Jaeger, Wurzburg, Germany) was used in all tests and calibrated daily using a 1 l syringe. The reference values were those recommended by the European Respiratory Society (ERS) [39]. However, since the ERS offers no reference values for ERV, the Jaeger Master Lab reference values were used instead. All tests were carried out between 9 am and 10 am with the subjects sitting in an upright position wearing a nose clip. No anti-obstructive medication was allowed prior to pulmonary function testing.

### Blood gas analyses

Arterial blood for the analysis of gases during room air breathing was drawn in all patients from the radial artery after a 5-min rest period. Arterial puncture was performed by either a physician (AMG) or two experienced nurses. For analyses we used an ABL 735 Radiometer (Copenhagen, Denmark) calibrated in accordance with the manufacturer’s specifications.

### Blood samples

Venous blood samples were collected after overnight fasting, clotted for 30 min at room temperature, and serum was separated by centrifugation. Samples were either analyzed immediately or stored at −80 °C until analysis [32]. NT-proBNP was analyzed by an immunometric assay on Vitros 5600 (Ortho-Clinical Diagnostics, NJ, USA), using calibrators and reagents from Ortho-Clinical Diagnostics according to the manufacturer’s instructions. Serum from the reference group had not been thawed previously. By contrast, 47 % of the baseline serum samples from the morbidly obese population had been thawed once, 5 % had been thawed two or three times, and 48 % had never been thawed. The frequency of freeze-thaw cycles did not differ significantly between the AHI groups or the intervention groups. The coefficient of variation was 8 %.

### Statistical analyses

Data are presented as mean (SD) or number (%) unless otherwise specified. Between-group comparisons were analyzed using independent samples *t* test, Mann–Whitney U test or multiple linear regression analysis for continuous variables and Fisher’s exact test for categorical variables. Within-group comparisons were performed using paired samples t test for continuous variables. The effect size and statistical significance of the uni- and multivariate associations were evaluated using Pearson’s correlation and linear regression analyses. NT-proBNP had a skewed distribution and the values were therefore transformed to approximate normal distribution using natural logarithms (ln-transformation). NT-proBNP values were missing in three subjects due to shortage of serum, and one subject had missing AHI value due to non-compliance. In addition, three subjects had predicted mean ΔNT-proBNP values >3 SD from mean and were excluded from the analyses (two of these subjects developed heart failure during the intervention period). The significance level was set at 0.05. Statistical analyses were performed using SPSS 22.0 (SPSS Inc., Chicago, IL).

## Results

A total of 76 subjects underwent laparoscopic Roux-en-Y gastric bypass surgery, [53 (70 %) female, mean (SD) age 43 (11) years, mean (SD) BMI 46.7 (5.7) kg/m^2^], while 63 subjects attended an intensive lifestyle intervention program, [44 (70 %) women, mean (SD) age 47 (11) years, mean (SD) BMI 43.4 (5.0) kg/m^2^]. Mean (SD) weight reduction was 30 (8) % in the surgery group and 8 (9) % in the lifestyle group [32] with post intervention BMIs of 32.6 (5.2) and 39.6 (5.6) kg/m^2^, respectively (p < 0.001).

### NT-proBNP—association with surgical and non-surgical weight loss

**Fig. 1:**
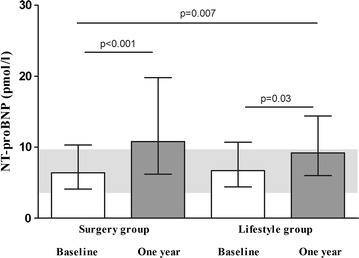
Median NT-proBNP-levels (25–75 percentiles) at baseline and after gastric bypass surgery (n = 71) and lifestyle intervention (n = 62). p values indicate significant changes within and between the intervention groups. The *grey background* represents the 25–75 percentile for the NT-proBNP levels in the normal weight healthy reference group (n = 30)

At baseline, women had significantly higher NT-proBNP levels than men [median (25–75 percentiles) 7.4 (5.2–12.3) pmol/l versus 5.1 (3.4–8.3) pmol/l, p = 0.004]. Moreover, NT-proBNP correlated significantly with age (r = 0.196, p = 0.024), but not with pack years (r = 0.002, p = 0.980). NT-proBNP at baseline did not differ between those who had gastric bypass surgery (n = 71) or those who were treated with intensive lifestyle intervention (n = 62, Fig. 1).

**Fig. 2:**
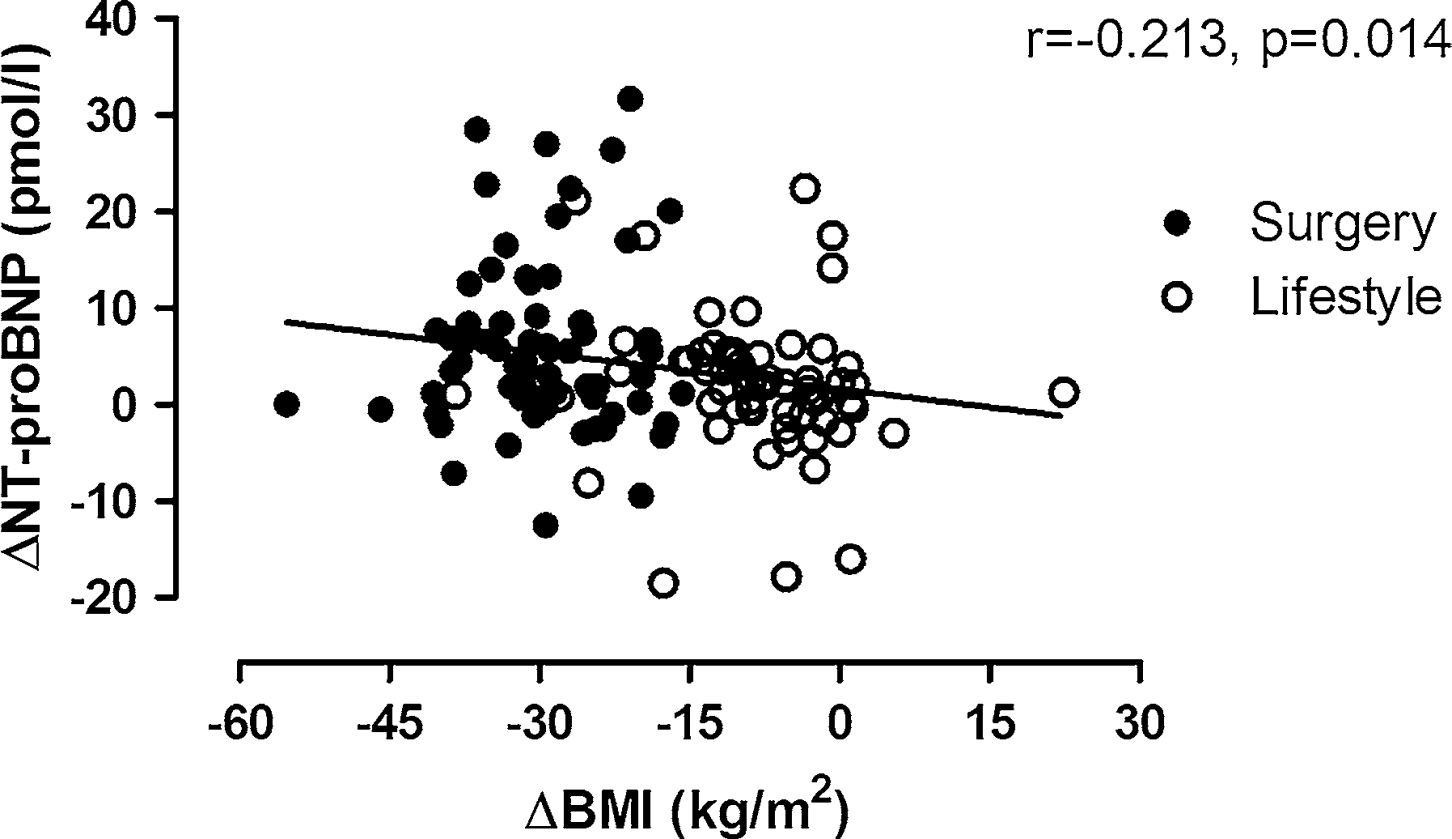
Scatter plot demonstrating the correlations between changes in NT-proBNP and changes in BMI after gastric bypass surgery and intensive lifestyle intervention

NT-proBNP increased significantly after both interventions, but significantly more in the surgery group (Fig. 1). The post intervention values were significantly higher in the surgery and lifestyle groups than the in the reference group (p < 0.001, p = 0.010, respectively). In addition, there was a negative correlation between changes in BMI and NT-proBNP (Fig. 2) and this correlation was independent of type of intervention (no “group x ΔNT-proBNP” interaction, p = 0.409).

### NT-proBNP at baseline—association with AHI and pulmonary function

Baseline characteristics and comorbidities of the low and high AHI groups are shown in Table 1 and Additional file 1: Table S1. In the low AHI group subjects were younger than in the high AHI group, while the mean BMI was lower and the proportion of females was higher. Four subjects (10 %) with high AHI had five or more central apneas during the night, whereas none with low AHI had central apneas (p = 0.008).

**Table 1:** Baseline characteristics according to AHI level

Variable	Low AHI (<15 events/hour)	High AHI (≥15 events/hour)	p
Number (n)	96	42	
Female gender (%)	76 (79)	20 (48)	<0.001
Age (years)	43.0 (11.1)	48.2 (9.4)	0.009
BMI (kg/m^2^)	44.4 (5.0)	46.8 (6.6)	0.022
Surgical treatment (%)	50 (52)	26 (62)	0.353
Arterial blood gases			
pO_2_ (kPa)	11.8 (1.3)	10.7 (1.4)	<0.001
pCO_2_ (kPa)	5.1 (0.5)	5.5 (0.5)	0.001
Diffusing capacity			
DLCO (diffusing capacity for carbon monoxide) (% pred)	93 (13)	92 (17)	0.752
DLCO/VA (diffusing capacity for CO/alveolar volume) (% pred)	106 (15)	111 (19)	0.135
Dynamic lung volumes			
FVC (forced vital capacity) (% pred)	104 (14)	98 (16)	0.055
FEV1 (forced vital capacity first second) (% pred)	94 (15)	93 (20)	0.680
Static lung volumes			
TLC (total lung capacity) (% pred)	100 (13)	94 (13)	0.033
IC (inspiratory capacity) (% pred)	123 (22)	115 (25)	0.098
VC (vital capacity) (% pred)	100 (15)	96 (18)	0.136
FRC (functional residual capacity) (% pred)	82 (17)	79 (16)	0.315
ERV (expiratory reserve volume) (% pred)	48 (35)	49 (34)	0.885
RV (residual volume) (% pred)	101 (28)	97 (27)	0.493

Data are presented as mean (SD), or n (%), as appropriate

**Fig. 3:**
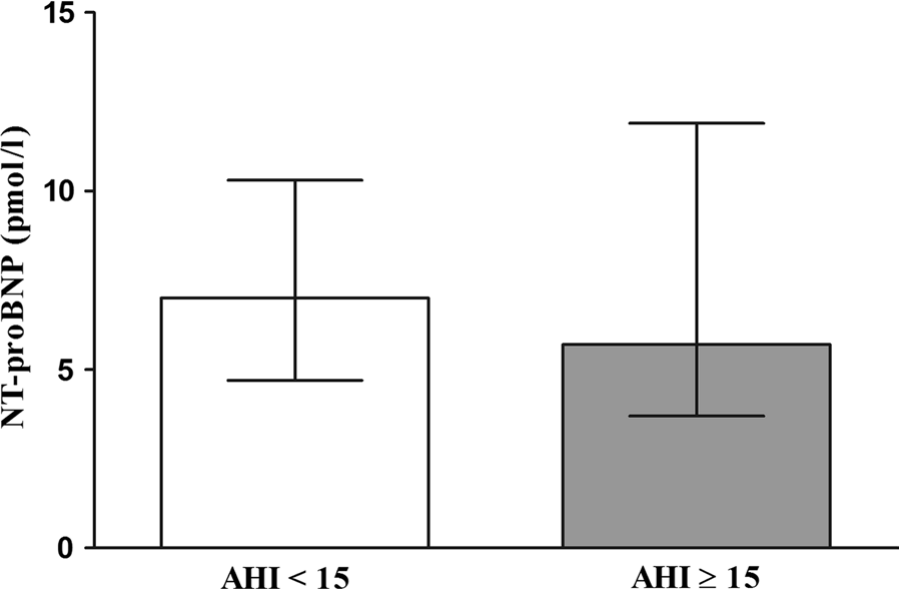
Median NT-proBNP-levels (25–75 percentiles) levels at baseline in subjects with AHI< and ≥15 events/hour

No significant difference in NT-proBNP levels at baseline between the low and high AHI groups were observed (Fig. 3). NT-proBNP correlated significantly and positively with both FRC (r = 0.329, p < 0.001) and RV (r = 0.215, p = 0.017), but not with other static or dynamic lung volumes, measures of diffusing capacity or blood gas values. The associations between NT-proBNP and FRC (Beta = 0.296, p = 0.002), but not RV (Beta = 0.152, p = 0.112), persisted after adjusting for pack years, gender, age and BMI.

### NT-proBNP during weight loss—association with changes in AHI and pulmonary function

**Fig. 4:**
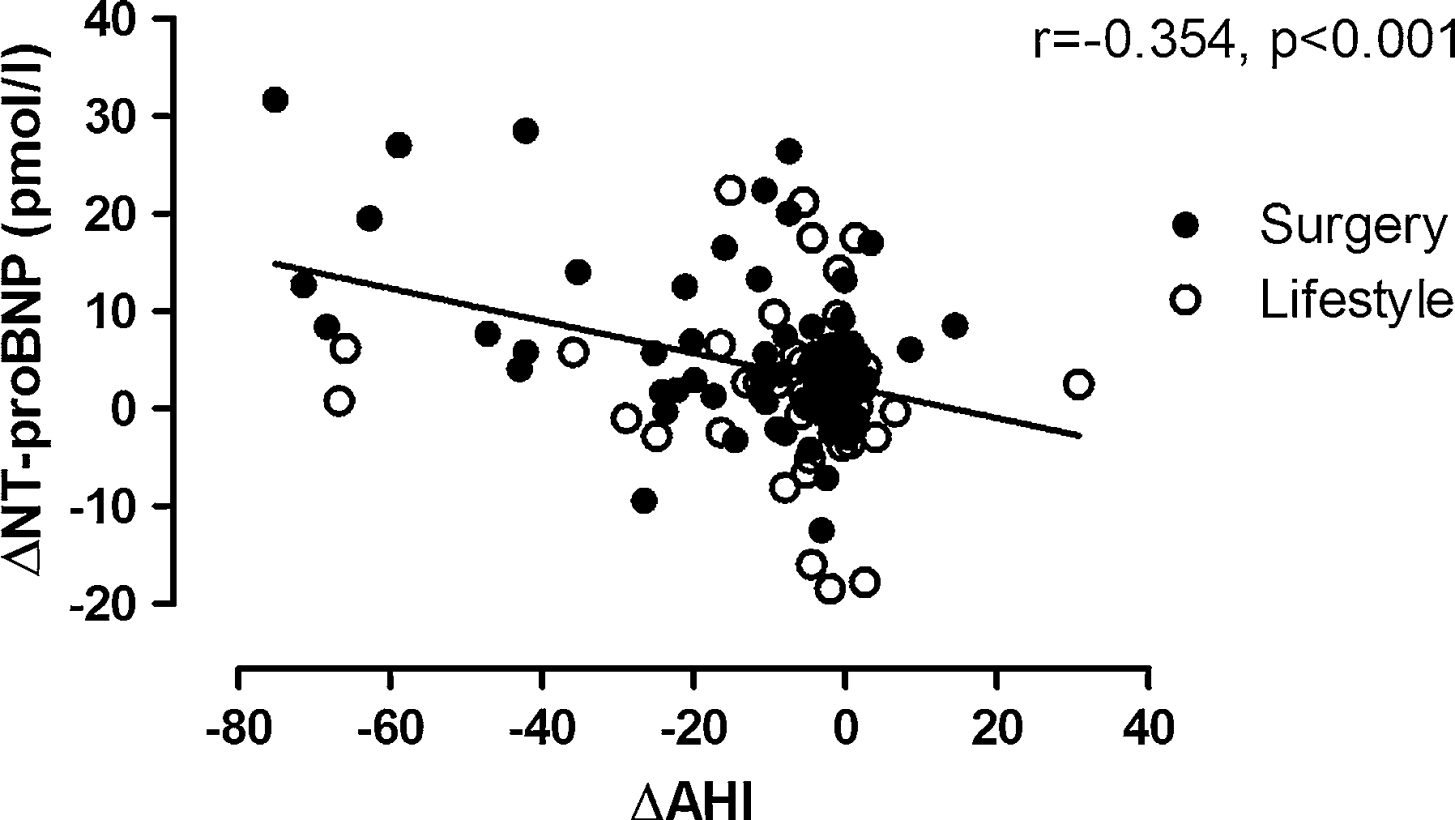
Scatter plot demonstrating the correlations between changes in NT-proBNP levels and changes in AHI after gastric bypass surgery and intensive lifestyle intervention

ΔAHI varied considerably (range −75 to 30 events/hour) and was negatively and significantly associated with ΔNT-proBNP (Fig. 4). This association persisted after adjusting for age, gender and ΔBMI (beta = −0.216, p = 0.021). In this model, the association between ΔBMI and ΔNT-proBNP observed in the univariate analyses was no longer significant (beta −0.130, p = 0.156). There was no significant interaction between treatment group and ΔAHI (p = 0.056). ΔNT-proBNP correlated significantly with ΔDLCO/VA (r = −0.185, p = 0.034), ΔIC (r = −0.225, p = 0.013) and ΔERV (r = 0.243, p = 0.007). However, after adjustments for age, gender, pack years and ΔBMI, these associations were attenuated and no longer significantly associated with ΔNT-proBNP.

### Pulmonary function—association with AHI at baseline and during weight loss

Hypercapnia and hypoxemia were observed in the high AHI group (Table 1). With the exception of a significantly higher TLC in the low AHI group, no differences in static and dynamic lung volumes or measures of diffusing capacity were observed. After adjustments for age, gender, pack years and baseline BMI, only pO_2_ remained significantly higher in subjects with AHI <15 than in those with AHI ≥15 (p = 0.035).

Univariate linear regression analyses showed that ΔAHI were negatively associated with changes in pO_2_, FVC, FEV1, VC and ERV and positively associated with changes in pCO_2_ (Additional file 1: Table S2). After multiple adjustments, the associations between ΔAHI and changes in pO_2_, pCO_2_, FVC and FEV1 were attenuated, but remained significant. Although statistically significant, the effect sizes were small and explained one to four percent of the variation (squared partial correlation) in these variables. By contrast, the associations between weight loss (ΔBMI) and dynamic lung volumes were large, explaining 25 % of the variation in both ΔFVC and ΔFEV1. No interactions with ΔBMI were found for any of these variables.

## Discussion

This study has three key findings. First, NT-proBNP values rose significantly more after gastric bypass surgery than after lifestyle intervention, and the post intervention NT-proBNP values in both groups were significantly higher than in a normal weight reference group. Second, enhanced NT-proBNP levels correlated with weight loss and reduction in AHI. Third, the results did not confirm our hypotheses that improved pulmonary function and improvement of OSA might counterbalance NT-proBNP-increase during weight loss.

In addition, reduced AHI was, independent of weight loss, associated with improved dynamic lung volumes and daytime blood gases. Together, these findings suggest that perturbed BNP release, indicated by low circulating NT-proBNP, could provide a common mechanism linking cardiometabolic disease and OSA in morbid obesity. Moreover, the study may have implications for the use of NT-proBNP as a clinical biomarker in such patients.

### NT-proBNP—associations with weight loss and AHI

Given the known obesity-associated increase in cardiac volume loading and left ventricular mass, the low to normal NT-proBNP values observed in morbid obesity in both the present study and previous studies [14, 15, 40] are unexpected. In fact, and in line with our finding, NT-proBNP increases after weight reduction induced by either gastric bypass [16, 17] or lifestyle interventions [18], while the heart is unloaded. In our study, patients following weight loss, while remaining overweight-obese, nevertheless exhibited higher NT-proBNP values compared to a normal weight reference group. This hitherto unknown phenomenon suggests that weight loss may unmask cardiac strain-induced BNP release. It is also possible that glomerular hyperfiltration which is associated with obesity [41, 42] may result in lower concentrations of NT-proBNP in obese patients. Weight loss also affects natriuretic peptide clearance mechanisms [21] such that systemic BNP availability is expected to increase further. These findings may indicate that the presence of obesity may disable BNP release, which could be regarded as safeguard against excess cardiac loading.

The association between sleep apnea and heart failure is well documented [43, 44]. NT-proBNP levels might therefore be expected to be particularly high in patients with OSA. However, our findings showed NT-proBNP levels to not be significantly higher in subjects with OSA than in subjects without OSA. Partly contrasting this finding, we found a negative association between changes in NT-proBNP and changes in AHI. The association remained after adjustments for changes in BMI. In partial agreement with this finding, an inverse association between obstructive sleep apnea severity and NT-proBNP levels was observed in a study of 1655 community-dwelling participants [45]. However, and in contrast to our findings, this association was markedly attenuated after adjustments for BMI. Furthermore, several other studies have reported conflicting data. In a community-based study of 349 Swedish women, a positive dose-relationship between the severity of AHI index and morning BNP level was found [46], and these findings were in line with an earlier, smaller study [47]. In a study of 64 patients with heart failure, no relationship between elevated BNP levels and frequency of sleep apnea was observed [48]. In another study including both patients with heart failure and healthy subjects, no associations between BNP levels and sleep apnoea severity were reported in either group [49].

To sum up, our findings did not support our hypothesis that reduced AHI might counterbalance NT-proBNP-increase during weight loss. Rather the contrary, the increase in NT-proBNP seemed to be more strongly associated with the reduction in AHI than the reduction in BMI.

### Pulmonary function—interplay with NT-proBNP and AHI

We have previously shown that improvements in blood gases and pulmonary function after surgical and non-surgical weight loss are mediated through weight loss [31]. The analyses presented in the current paper go further and show significant associations between decreased AHI and improved blood gases as well as dynamic volumes. A decrease in obstructive sleep apnea severity was, independent of weight loss, associated with improvements in arterial pO_2_ and pCO_2_ as well as an increase in FEV1 and FVC. It has been shown that natriuretic peptide receptors are expressed in the lung, and natriuretic peptide infusion elicits bronchial dilatation [29]. It is tempting to speculate that natriuretic peptides in this way may improve lung function and thereby possibly explain the weight loss independent association between increasing NT-proBNP and decreasing AHI. Importantly, with the exception of a small positive independent association between NT-proBNP and FRC at baseline, we did not find any significant associations between NT-proBNP levels and measures of pulmonary function at baseline or during weight loss supporting this hypothesis.

## Strengths and limitations

The strengths of this study include the prospective and comparative design, and the generalizability of the study is relatively high due to the small number of exclusion criteria. The non-randomized design reduces the internal validity of the study. The serum samples were frozen in a biobank for up to 8 years. Although NT-proBNP has been shown to be stable after storage frozen for 2 years and after multiple freeze-thaw cycles [50, 51], we cannot rule out that the higher frequency of freeze-thaw cycles in the morbidly obese population might have resulted in lower NT-proBNP levels than in the reference population. Due to multiple comparisons there is an increased risk of false positive results (type 1 errors). Echocardiography pre and post intervention might have added important information regarding heart function. In addition, the present study lacks compliance data from the CPAP and bilevel machines regarding the use at night. Finally, the study participants were mainly of European origin, meaning that the results cannot automatically be generalized to include other ethnic groups.

## Conclusions

In conclusion, weight loss following life style intervention or bariatric surgery reverses BNP deficiency in patients with morbid obesity. This increase could beneficially affect cardiovascular and metabolic disease. More research regarding the beneficial effects of NT-proBNP and BNP in heart failure is thus needed, a position supported by findings from the PARADIGM study which showed improved outcomes in heart failure patients treated with combined angiotensin receptor and neprilysin inhibition [52, 53]. The latter augments systemic natriuretic peptide availability.

In contrast with our hypotheses, the significant improvements in OSA and pulmonary function at follow up did not counterbalance the NT-proBNP-increase during weight loss. Rather the contrary, the increase in NT-proBNP seemed to be more strongly associated with the reduction in AHI than the reduction in BMI. This is difficult to explain, but one might speculate that natriuretic peptides may elicit bronchial dilatation [29] and, accordingly, improve lung function and OSA. However, our findings are clearly hypothesis generating and need further study.

## Additional file

10.1186/s13104-016-2241-x Additional tables.

## Abbreviations

BMI: body mass index
NT-proBNP: N-terminal peptide
OSA: obstructive sleep apnea
AHI: apnea hypopnea index
CPAP: continuous positive airway pressure
FEV1: forced expiratory volume in one second
FVC: forced vital capacity
DLCO: diffusing capacity for carbon monoxide
DLCO/VA: diffusing capacity for carbon monoxide/alveolar volume
TLC: total lung capacity
IC: inspiratory capacity
VC: vital capacity
FRC: functional residual capacity
ERV: expiratory reserve volume
RV: residual volume
