# Supplementary material for: The effect of surgical and non-surgical weight loss on N-terminal pro-B-type natriuretic peptide and its relation to obstructive sleep apnea and pulmonary function
Source: BMC Res Notes. 2016 Sep 13;9(1):440. doi: 10.1186/s13104-016-2241-x (PMC5020450; doi:10.1186/s13104-016-2241-x)
Supplement: Supplementary file 1 — 10.1186/s13104-016-2241-x Additional tables. [file 13104_2016_2241_MOESM1_ESM.docx]

**Additional file 1: Gabrielsen AM et al. The effect of surgical and non-surgical weight loss on N-terminal pro-B-type natriuretic peptide and its relation to obstructive sleep apnea and pulmonary function**

| **Table S1.** Comorbidities at baseline according to AHI level | | | |
| --- | --- | --- | --- |
| **Variable** | **Low AHI**  **(< 15 events/hour)** | **High AHI**  **(≥ 15 events/hour)** | **P** |
| Number (n) | 96 | 42 |  |
| Hypertension (%) | 50 (52) | 30 (71) | 0.040 |
| Coronary disease (%) | 3 (3.1) | 6 (14.3) | 0.023 |
| Atrial fibrillation (%) | 1 (1.0) | 0 (0) | 1.000 |
| Type 2 diabetes (%) | 21 (21.9) | 16 (38.1) | 0.061 |
| Asthma (%) | 31 (32.3) | 10 (23.8) | 0.419 |
| Antiobstructive medication (%) | 32 (33.3) | 12 (28.6) | 0.692 |
| Chronic obstructive pulmonary disease (%) | 5 (5.2) | 4 (9.5) | 0.455 |
| Pack-years | 9.5 (14) | 10.5 (14) | 0.694 |
| Continuous positive airway pressure (%) | 21 (21.9)^.^ | 29 (69.0) | <0.001 |
| Bilevel continuous airway pressure (%) | 2 (2.1) | 11 (26.8) | <0.001 |
| Apnea/hypopnea index | 5.6 (4.3) | 42.7 (22.0) | <0.001 |
| Oxygen desaturation index | 7.2 (6.0) | 40.3 (22.8) | <0.001 |
| Data are presented as mean (SD), or n (%), as appropriate. | | | |

| **Table S2.** The associations between changes in AHI and BMI and changes in blood gases and measures of pulmonary function | | | | | |
| --- | --- | --- | --- | --- | --- |
| **Dependent variables** | **Independent variables** | **Unadjusted beta** | **P** | **Adjusted beta** | **P** |
| ∆ BMI (kg/m²) | ∆ AHI | 0.273 | 0.001 | 0.292 | 0.001 |
| ∆ pO_2_ (kPa) | ∆ AHI  ∆ BMI | -0.322  -0.265 | <0.001  0.002 | -0.204  -0.216 | 0.019  0.011 |
| ∆ pCO_2_ (kPa) | ∆ AHI  ∆ BMI | 0.199  -0.053 | 0.022  0.069 | 0.199  -0.086 | 0.035  0.345 |
| ∆ HCO3 (kPa) | ∆ AHI  ∆ BMI | 0.163  -0.097 | 0.063  0.260 | 0.154  -0.126 | 0.098  0.165 |
| ∆ FVC (l) | ∆ AHI  ∆ BMI | -0.301  -0.532 | <0.001  <0.001 | -0.168  -0.489 | 0.028  <0.001 |
| ∆ FEV1(l) | ∆ AHI  ∆ BMI | -0.281  -0.501 | 0.001  <0.001 | -0.160  -0.449 | 0.042  <0.001 |
| ∆ DLCO(mmol/kPA/l) | ∆ AHI  ∆ BMI | -0.059  -0.038 | 0.502  0.662 | -0.052  -0.017 | 0.571  0.855 |
| ∆ DLCO/VA | ∆ AHI  ∆ BMI | 0.097  0-476 | 0.268  <0.001 | -0.035  0.481 | 0.666  <0.001 |
| ∆ TLC (l) | ∆ AHI  ∆ BMI | -0.065  -0.206 | 0.471  0.020 | -0.012  -0.197 | 0.898  0.038 |
| ∆ IC (l) | ∆ AHI  ∆ BMI | 0.114  0.497 | 0.204  <0.001 | -0.041  0.509 | 0.619  <0.001 |
| ∆ VC (l) | ∆ AHI  ∆ BMI | -0.264  -0.498 | 0.003  <0.001 | -0.124  -0.456 | 0.137  <0.001 |
| ∆ FRC (l) | ∆ AHI  ∆ BMI | -0.161  -0.606 | 0.074  <0.001 | 0.016  -0.606 | 0.840  <0.001 |
| ∆ ERV (l) | ∆ AHI  ∆ BMI | -0.246  -0.632 | 0.006  <0.001 | -0.063  -0.607 | 0.398  <0.001 |
| ∆ RV (l) | ∆ AHI  ∆ BMI | 0.115  0.117 | 0.204  0.190 | 0.077  0.089 | 0.414  0.344 |
| Pulmonary function tests are adjusted for ΔBMI and packyears. Blood gases values were in addition adjusted for age and gender. Abbreviations: DLCO, diffusing capacity for carbon monoxide; DLCO/VA, diffusing capacity for CO/ alveolar volume; FVC, forced vital capacity; FEV1, forced vital capacity first second; FEV1, forced vital capacity first second; TLC, total lung capacity; IC, inspiratory capacity; VC, vital capacity; FRC, functional residual capacity; ERV, expiratory reserve volume; RV, residual volume. | | | | | |
